# Increased MFG‐E8 at neuromuscular junctions is an exacerbating factor for sarcopenia‐associated denervation

**DOI:** 10.1111/acel.13536

**Published:** 2021-12-24

**Authors:** Madoka Ikemoto‐Uezumi, Heying Zhou, Tamaki Kurosawa, Yuki Yoshimoto, Masashi Toyoda, Nobuo Kanazawa, Tatsu Nakazawa, Mitsuhiro Morita, Kunihiro Tsuchida, Akiyoshi Uezumi

**Affiliations:** ^1^ Muscle Aging and Regenerative Medicine Tokyo Metropolitan Institute of Gerontology (TMIG) Tokyo Japan; ^2^ Laboratory of Veterinary Pharmacology Department of Veterinary Medical Sciences Graduate School of Agriculture and Life Sciences Tokyo University Tokyo Japan; ^3^ Vascular Medicine TMIG Tokyo Japan; ^4^ Department of Surgery Tokyo Metropolitan Geriatric Hospital and Institute of Gerontology Tokyo Japan; ^5^ Seibo Hospital Tokyo Japan; ^6^ Department of Orthopaedic Surgery Fujita Health University Toyoake Japan; ^7^ Division for Therapies against Intractable Diseases Institute for Comprehensive Medical Science Fujita Health University Toyoake Japan

**Keywords:** aging, denervation, MFG‐E8, neuromuscular junction, sarcopenia, skeletal muscle

## Abstract

Sarcopenia is an important health problem associated with adverse outcomes. Although the etiology of sarcopenia remains poorly understood, factors apart from muscle fibers, including humoral factors, might be involved. Here, we used cytokine antibody arrays to identify humoral factors involved in sarcopenia and found a significant increase in levels of milk fat globule epidermal growth factor 8 (MFG‐E8) in skeletal muscle of aged mice, compared with young mice. We found that the increase in MFG‐E8 protein at arterial walls and neuromuscular junctions (NMJs) in muscles of aged mice. High levels of MFG‐E8 at NMJs and an age‐related increase in arterial MFG‐E8 have also been identified in human skeletal muscle. In NMJs, MFG‐E8 is localized on the surface of terminal Schwann cells, which are important accessory cells for the maintenance of NMJs. We found that increased MFG‐E8 at NMJs precedes age‐related denervation and is more prominent in sarcopenia‐susceptible fast‐twitch than in sarcopenia‐resistant slow‐twitch muscle. Comparison between fast and slow muscles further revealed that arterial MFG‐E8 can be uncoupled from sarcopenic phenotype. A genetic deficiency in MFG‐E8 attenuated age‐related denervation of NMJs and muscle weakness, providing evidence of a pathogenic role of increased MFG‐E8. Thus, our study revealed a mechanism by which increased MFG‐E8 at NMJs leads to age‐related NMJ degeneration and suggests that targeting MFG‐E8 could be a promising therapeutic approach to prevent sarcopenia.

## Graphical abstract

Milk fat globule epidermal growth factor 8 (MFG‐E8) levels at neuromuscular junctions (NMJs) increase during aging. A genetic deficiency in MFG‐E8 attenuated age‐related denervation of NMJs and muscle weakness, providing evidence of a pathogenic role of increased MFG‐E8. This study revealed a previously unrecognized mechanism of age‐related NMJ denervation associated with sarcopenia.

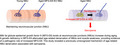

## 1 INTRODUCTION

Sarcopenia, the loss of skeletal muscle mass and strength with age, is a progressive and generalized disease in older adults. Weakened muscles increase risk of falls and fractures, which can cause frailty, loss of independence, and even mortality. Although suppressing sarcopenia is important, its pathogenesis is multifactorial and remains poorly understood. A decline in muscle strength precedes the loss of muscle mass in older adults, suggesting that decreased muscle quality is a causal factor of sarcopenia (Goodpaster et al., 2006). Venturelli et al. (2015) examined the impact of advanced aging and disuse on skeletal muscle function by measuring contractile properties in vivo and in vitro in the lower limbs of young (aged 25 ± 6 years), oldest‐old mobile (aged 87 ± 5 years), and immobile (aged 88 ± 4 years) women. The study indicated that neither advanced aging nor disuse, per se, impacts intrinsic skeletal muscle function assessed in vitro. However, muscle function in vivo is attenuated by age and exacerbated by disuse, implying that a combination of factors apart from muscle fibers, such as muscle innervation and the composition of the extracellular matrix (ECM), diminishes function (Grounds & Pinniger, 2015; Venturelli et al., 2015, 2018). Studies of parabiosis have indicated that changes in humoral factors during aging are also related to the age‐related deterioration of tissue function (Zhang et al., 2019).

Age‐related changes in neuromuscular junctions (NMJs) are one of the most typical deteriorations in non‐myofiber components associated with sarcopenia. Age‐related declines in function and morphological changes occur in NMJs (Badawi & Nishimune, 2020), which are synapses formed between the presynaptic motor nerve terminals and the postsynaptic myofiber membranes. These synapses enable the transmission of neurotransmitters from motor nerves to myofiber membranes and are thus essential for muscle contraction and function. Aging leads to the fragmentation of postsynaptic acetylcholine receptor (AChR) clusters and progressive denervation, the withdrawal of nerve terminals from NMJs (Chai et al., 2011; Deschenes et al., 2010; Lexell & Downham, 1991; Mosole et al., 2014; Valdez et al., 2010). Denervation at the NMJ precedes the degradation of spinal motor neurons, which is referred to as, “dying back” neuropathy (Chai et al., 2011; Chung et al., 2017). Two types of accessory cells are found in NMJs. Terminal Schwann cells (tSCs) cover NMJs, and function in their formation, maintenance, and regeneration (Barik et al., 2016; Li et al., 2018). The tSCs are non‐myelinating glial cells, unlike myelinating SCs that cover motor nerve axons. A recent specific labeling study of tSCs showed that they have cellular and molecular features that differ from those of myelinating SCs (Castro et al., 2020). Aging leads to tSC degeneration (Ludatscher et al., 1985), and decreased numbers of tSCs and ratios (%) of NMJs containing them (Snyder‐Warwick et al., 2018). Such changes in tSCs with age may contribute to NMJ denervation. Interstitial mesenchymal progenitors, also known as fibro/adipogenic progenitors (Joe et al., 2010; Uezumi et al., 2010), comprise another accessory cell type that are necessary for NMJ maintenance. We recently located mesenchymal progenitors adjacent to Schwann cells in mice and found that they surround motor nerve axons, and cover NMJs (Uezumi et al., 2021). We showed that mesenchymal progenitors play an important role in maintaining NMJs by stabilizing Schwann cell characteristics via Bmp3b (Uezumi et al., 2021). Despite these advances in understanding the maintenance of NMJs, the precise mechanisms of age‐related NMJ degeneration are not fully understood.

Milk fat globule epidermal growth factor 8 (MFG‐E8) is a secreted glycoprotein that participates in various cellular functions, including the phagocytosis of apoptotic cells (Hanayama et al., 2002, 2004; Raymond et al., 2009), maintenance of neural stem cell pool (Okazaki & Gotoh, 2018; Zhou et al., 2018), the proliferation of vascular smooth muscle cells (Wang et al., 2012), sperm‐egg binding (Ensslin & Shur, 2003), and the development of mammary glands (Ensslin & Shur, 2007). Rodent MFG‐E8 has long and short isoforms derived by alternative splicing. The short form is more abundantly and ubiquitously expressed than the long form (Aziz et al., 2011). Only the short isoform has been identified in humans (Wang et al., 2013). Levels of MFG‐E8 mRNA and protein increase in aged, atherosclerotic, hypertensive, and diabetic arterial walls of several mammalian species, including humans (Fu et al., 2009; Wang et al., 2013). Thus, MFG‐E8 might play a key role in the pathogenesis of chronic arterial inflammation (Wang et al., 2013, 2014). The cleavage of MFG‐E8 produces a short, internal, 50‐amino acid fragment form called medin (Häggqvist et al., 1999; Peng et al., 2005), which is the most common form of human amyloid. It is located mainly in the arteries of the upper body, that is, thoracic aorta and temporal artery, in almost all humans aged >50 years (Mucchiano et al., 1992; Peng et al., 2005). Degenhardt et al. (2020) recently revealed that a genetic deficiency of MFG‐E8 eliminates vascular aggregates and prevents the age‐related decline of cerebrovascular function in mice, suggesting that age‐related MFG‐E8 aggregation causes cerebrovascular dysfunction.

This study comprehensively examined age‐related changes in humoral factors in skeletal muscle to identify the factors involved in sarcopenia. We found that MFG‐E8 is the most prevalent upregulated factor in aged muscle. We revealed the increase in the amount of MFG‐E8 not only in arteries but also at NMJs in skeletal muscle as age advances. The increase of MFG‐E8 in skeletal muscle occurs before the age‐related loss of muscle mass and denervation. The levels of MFG‐E8 and the rate of denervation are high among NMJs in sarcopenia‐susceptible fast‐twitch muscle, but low among those in sarcopenia‐resistant slow‐twitch muscle. Furthermore, the pathological relevance of increased MFG‐E8 has been proven by the attenuated denervation and muscle weakness of aged MFG‐E8 deficient mice. Our findings indicated that MFG‐E8 is an aggravating factor for the age‐related NMJ denervation and muscle weakness, and thus, it could be a promising molecular target for sarcopenia prevention.

## 2 RESULTS

### 2.1 Increased levels of MFG‐E8 protein in skeletal muscle of aged mice

**FIGURE 1:**
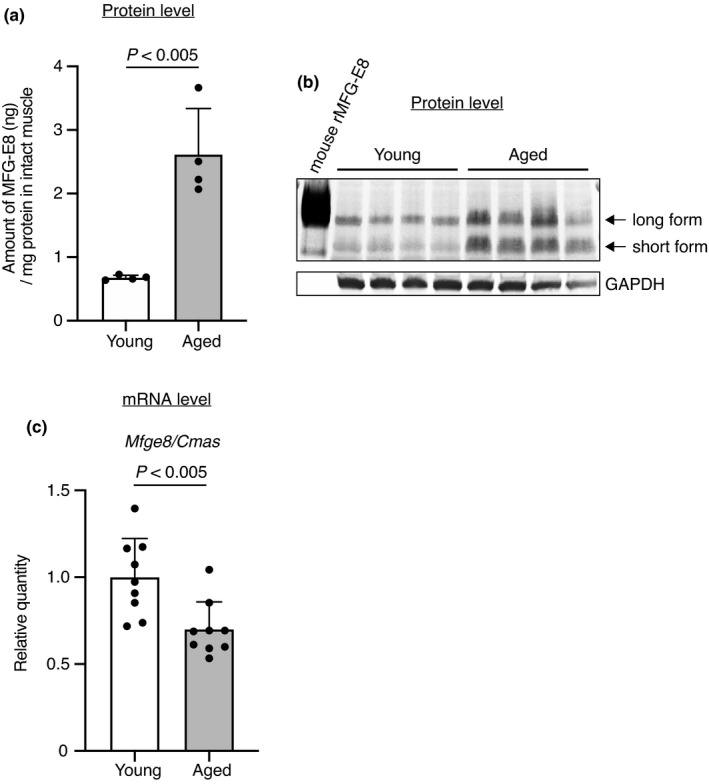
Increased MFG‐E8 protein in skeletal muscle of aged mice. (a) ELISA and (b) immunoblot of MFG‐E8 in tibialis anterior (TA) muscles of young (aged 10–12 weeks) and aged mice (aged 25–26 months). *n* = 4 per group. ELISA data represent the means ± *SD*; two‐sided unpaired *t* tests. Internal control of immunoblot is GAPDH. (c) Expression of *Mfge8* mRNA in TA muscles of young (aged 9–12 weeks) and aged mice (aged 25–28 months) determined in triplicate by real‐time quantitative PCR. Raw values of *Mfge8* were normalized to that of *Cmas*. *n* = 9 per group. Data represent the means ± *SD*; two‐sided unpaired *t* tests

To examine age‐related changes in humoral factors, we performed cytokine antibody array in intact skeletal muscles of young and aged (3 and 25 months, respectively) mice. Table 1 shows the factors that were upregulated ≥1.5‐fold among 144 cytokine‐related factors in aged mice, compared with young mice. Downregulated factors (≤0.65) in aged mice were undetectable. We focused on MFG‐E8, the expression of which was the highest in aged, compared with young muscle (Table 1). We examined age‐related changes in the amount of MFG‐E8 protein using an ELISA and confirmed that MFG‐E8 was significantly increased 3.8‐fold in the muscle of aged, compared with young mice (Figure 1a). Rodents have long and short isoforms of MFG‐E8 derived from alternative splicing (Wang et al., 2013), both of which were remarkably increased in aged muscles (Figure 1b). In contrast to protein levels, *Mfge8* mRNA expression was significantly decreased in muscles of aged mice compared with young mice (Figure 1c).

**TABLE 1:** Cytokines with altered expression in intact skeletal muscles of aged mice

Cytokine	Fold change (Aged vs. Young)
Upregulated ≥1.5‐fold	
MFG‐E8	3.04
bFGF	2.25
IL‐6R	2.00
Fc gamma receptor IIB	1.99
ICAM‐1	1.68
PF‐4 (CXCL4)	1.57
Cardiotrophin‐1	1.53
SHH‐N (Sonic hedgehog N‐product)	1.52
Axl (AXL receptor tyrosine kinase)	1.52
Downregulated ≤0.65‐fold	N.D.

Note

Cytokine antibody array in tibialis anterior (TA) muscles of young (aged 12 weeks) and aged mice (aged 25 months). *n* = 2 per group. N.D.; not detectable.

### 2.2 Specific increase in MFG‐E8 protein levels at artery walls and neuromuscular junctions in skeletal muscles of aged mice

**FIGURE 2:**
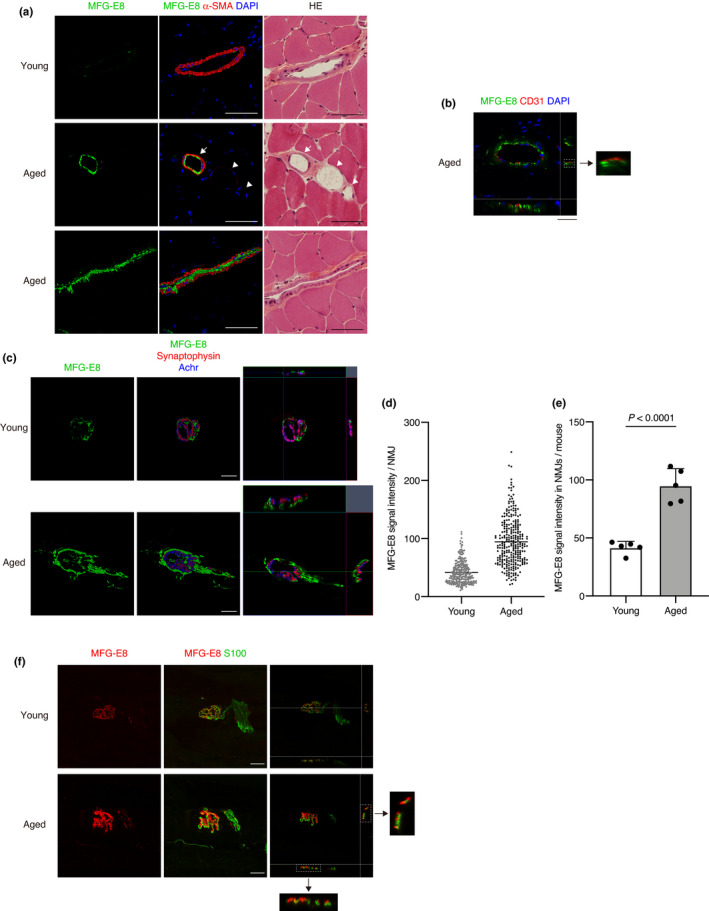
Increased MFG‐E8 protein in arteries and NMJs in skeletal muscles of aged mice. (a) Immunostaining of TA from young (aged 3 months) and aged mice (aged 27–28 months) for MFG‐E8 and α‐smooth muscle actin (α‐SMA). Nuclei were stained with DAPI. After processing for immunofluorescence staining, muscle sections were stained with hematoxylin and eosin (HE). Arrow, artery; Arrowheads, veins. (b) Immunostaining of TA from aged mouse (aged 24 months) for MFG‐E8 and CD31. Nuclei were stained with DAPI. A magnified image of boxed region shows MFG‐E8 localization on the surface of CD31(+) endothelial cells. (c) Whole‐mount immunostaining of extensor digitorum longus (EDL) muscles of young (aged 3 months) and aged mice (aged 25 months) for MFG‐E8, synaptophysin, and acetylcholine receptor (Achr). (d) Quantification of MFG‐E8 signal intensity per NMJ in TA. Total 271 NMJs from five young mice (aged 9–12 weeks) and total 284 NMJs from five aged mice (aged 24–28 months) are quantified. Data represent the values for each NMJ and the means. (e) Quantification of MFG‐E8 signal intensity at NMJs in TA per mouse. Young (aged 9–12 weeks) and aged mice (aged 24–28 months). *n* = 5 per group. Data represent the means ± *SD*; two‐sided unpaired *t* tests. (f) Immunostaining of TA of young (aged 3 months) and aged mice (aged 24 months) for MFG‐E8 and S100. Magnified images of boxed regions show MFG‐E8 localization on the surface of S100(+) terminal Schwann cells. Scale bars: 50 µm (a), 25 µm (b, c, and f)

We immunohistochemically localized MFG‐E8 in skeletal muscle. We detected MFG‐E8 in the inner walls of arteries characterized by a thick α‐smooth muscle actin (α‐SMA) layer, but not in veins of aged mice (Figure 2a). In contrast, MFG‐E8 was undetectable in arteries of young muscle (Figure 2a). One study found that MFG‐E8 is expressed in arterioles of mouse hindlimb muscles (Silvestre et al., 2005), but age‐related changes in MFG‐E8 in the lower extremities remained unknown. Double immunostaining of MFG‐E8 and CD31 revealed the localization of MFG‐E8 on the surface of endothelial cells (Figure 2b). In addition to arteries, we found that MFG‐E8 is also localized in NMJs and significantly increased in aged muscles (Figure 2c). The signal intensity of MFG‐E8 in NMJs of aged mice was significantly higher than that of young mice (Figure 2d,e). To our knowledge, MFG‐E8 has not been recognized in NMJs until now. We localized MFG‐E8 at the presynaptic side with synaptophysin, but not in the postsynaptic side with acetylcholine receptor (Achr) (Figure 2c). Further investigation of the localization of MFG‐E8 revealed that it is present in large amounts on the surface of aged tSCs (Figure 2f). These results indicate that age‐related increase in MFG‐E8 protein in skeletal muscle occurs specifically at the arterial walls and tSCs.

### 2.3 Localization of MFG‐E8 in arteries and NMJs of human skeletal muscle

**FIGURE 3:**
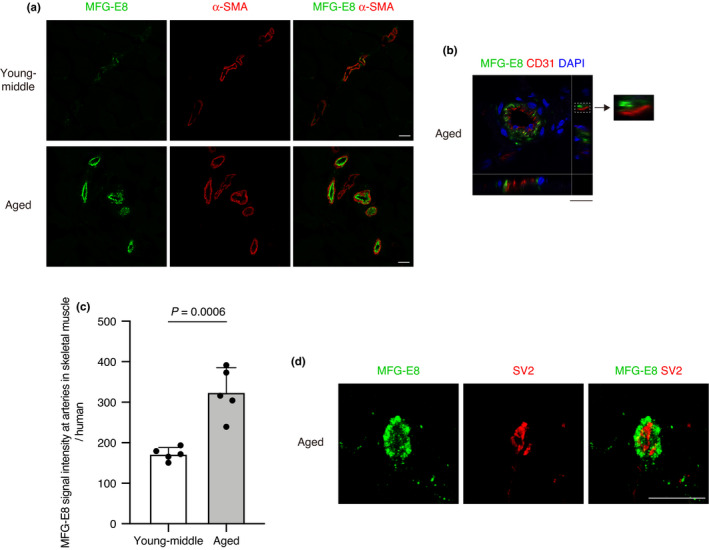
Localization of MFG‐E8 at artery and NMJs in skeletal muscle of aged human. (a) Immunostaining of gluteus medius muscle of young‐middle and aged humans for MFG‐E8 and α‐SMA. (b) Immunostaining of gluteus medius muscle of aged human for MFG‐E8 and CD31. Nuclei were stained with DAPI. A magnified image of boxed region shows MFG‐E8 localization on the surface of CD31(+) endothelial cells. (c) Quantification of MFG‐E8 signal intensity at arteries in gluteus medius per human. Young‐middle (aged 30–48 years) and aged humans (aged 73–85 years). *n* = 5 per group. Data represent the means ± *SD*; two‐sided unpaired *t* tests. (d) Immunostaining of TA muscle of aged human (representative data of age 90 patient) for MFG‐E8 and synaptic vesicle glycoprotein 2 (SV2). Scale bars: 50 µm (a), 25 µm (b, d)

We explored whether MFG‐E8 is expressed in human skeletal muscle. Like the results obtained from mice, high levels of MFG‐E8 protein were found at arterial walls in the gluteus medius of humans aged 73–85 years, but barely detectable in humans aged 30–48 years (Figure 3a). Arterial MFG‐E8 was localized on the surface of endothelial cells as observed in mice (Figure 3b). The signal intensity of MFG‐E8 at arteries in gluteus medius of aged human was significantly higher than that of young‐middle human (Figure 3c). Other researchers have found that MFG‐E8 is expressed in blood vessels of the upper body, including the thoracic aorta and temporal artery, in virtually all humans aged >50 years (Mucchiano et al., 1992; Peng et al., 2005). However, its expression in the blood vessels of lower human extremities has remained unknown. We also found conspicuous levels of MFG‐E8 protein at NMJs of humans aged 64–90 years (Figure 3d). The NMJs comprise only a small part of skeletal muscle, which hampers their identification in small pieces of muscle obtained from biopsy specimens. Therefore, we used a relatively large volume of skeletal muscle obtained by surgical amputation of a lower extremity to examine NMJs in aged humans, as described (Jones et al., 2017). Because we were unable to obtain a sufficient volume of muscle from young humans, we could not compare levels of MFG‐E8 protein at NMJs between young and aged humans in this study. However, at the very least, we could detect conspicuous levels of MFG‐E8 protein at NMJs of aged human muscle.

### 2.4 The increase in MFG‐E8 at NMJs precedes age‐related loss of muscle mass and NMJ denervation

**FIGURE 4:**
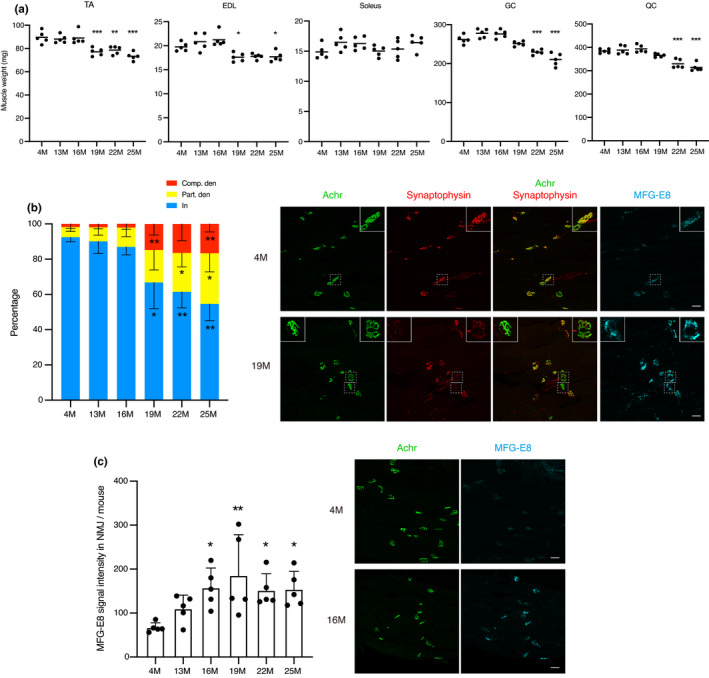
Time course changes of muscle weight, NMJ status, and MFG‐E8 protein levels in NMJs. (a) Time course changes of muscle weight from 4‐ to 25‐month‐old. Weight of each muscle is shown as sum of both hindlimbs. *n* = 5 mice in each time point. Data represent the values for each mouse and the means. ANOVA followed by Dunnett's tests; *p < 0.05, **p < 0.01, ***p < 0.001 vs. 4 M. (b) Time course changes of ratios of completely (Comp. den) and partially (Part. den) denervated, and innervated (In) NMJs in TA muscle. *n* = 5 mice in each time point. Data represent the means ± *SD*; ANOVA followed by Dunnett's tests; *p < 0.05, **p < 0.01 vs. 4 M. Immunostaining of TA muscles of 4‐ and 19‐month‐old mice for Achr, synaptophysin, and MFG‐E8. Insets show magnified images of boxed regions. Insets in upper panels show innervated (In) NMJ. Left and right insets in lower panels show completely (Comp. den) and partially (Part. den) denervated NMJ, respectively. (c) Time course changes of MFG‐E8 signal intensity at NMJs in TA muscle per mouse. *n* = 5 mice in each time point. Data represent the means ± *SD*; ANOVA followed by Dunnett's tests; *p < 0.05, **p < 0.01 vs. 4 M. Immunostaining of TA muscles of 4‐ and 16‐month‐old mice for Achr and MFG‐E8. Scale bars: 50 µm (b, c). TA, tibialis anterior; EDL, extensor digitorum longus; GC, gastrocnemius; QC, quadriceps

We next investigated the interrelationship among muscle weight, NMJ status, and MFG‐E8 levels at NMJs during the aging process. The weights of tibialis anterior (TA), extensor digitorum longus (EDL), soleus, gastrocnemius (GC), and quadriceps (QC) muscles were measured. The loss of muscle weight was evident in TA and EDL muscles by 19 months of age and GC and QC muscles by 22 months, but not observed in soleus muscle (Figure 4a). Various structural alterations in NMJs, including partial or complete withdrawal of axons from some postsynaptic sites, occur during aging. Time course investigation of NMJ status in TA mouse muscle revealed that denervated NMJs significantly increase by 19 months (Figure 4b), which is consistent with a previous study (Valdez et al., 2010). Importantly, MFG‐E8 protein levels at NMJs in TA muscle significantly increased as early at 16 months of age in the same cohort of mice (Figure 4c). Therefore, increased MFG‐E8 protein at NMJs precedes loss of muscle weight and denervation of NMJs. The temporal relationship among muscle weight, NMJ status, and MFG‐E8 levels at NMJs suggests that the increase in MFG‐E8 at NMJs is a contributing factor to age‐related mouse muscle mass loss and NMJ denervation.

### 2.5 The increase in MFG‐E8 at NMJs is more prominent in fast‐twitch muscle than in slow‐twitch muscle

**FIGURE 5:**
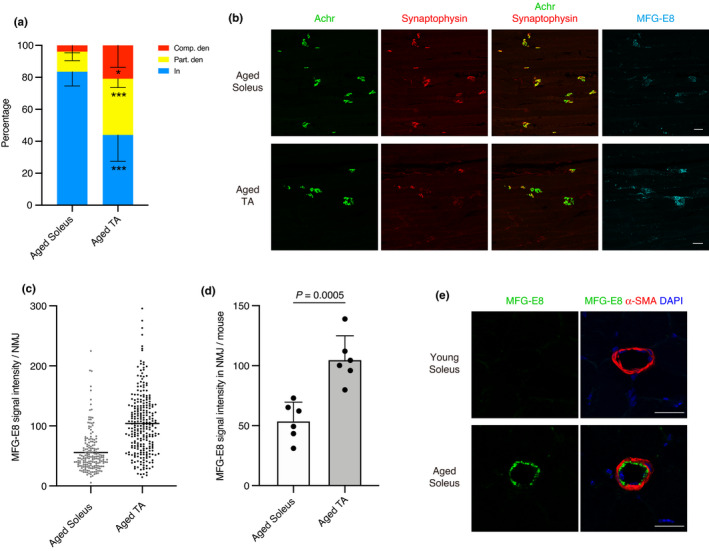
NMJ denervation and the increase of MFG‐E8 protein are more prominent in fast‐, than slow‐twitch muscle. (a) Ratios of completely (Comp. den) and partially (Part. den) denervated, and innervated (In) NMJ in soleus and TA muscles of aged mice (aged 24–29 months). *n* = 6 mice. Data represent the means ± *SD*; two‐sided unpaired *t* tests; *p < 0.05, ***p < 0.001. (b) Soleus and TA muscles of same mouse (aged 29 months) were immunostained for Achr, synaptophysin, and MFG‐E8. Note that NMJs in soleus muscle were scarcely stained for MFG‐E8. (c) Quantification of MFG‐E8 signal intensity per NMJ in soleus and TA muscles. Total 185 NMJs for soleus and total 259 NMJs for TA from six mice (aged 24–29 months) are quantified. Data represent the values for each NMJ and the means. (d) Quantification of MFG‐E8 signal intensity at NMJs in soleus and TA per mouse (aged 24–29 months). *n* = 6 mice. Data represent the means ± SD; two‐sided unpaired *t* tests. (e) Immunostaining of soleus muscles from young (aged 6 months) and aged mice (aged 24 months) for MFG‐E8 and α‐SMA. Nuclei were stained with DAPI. Scale bars: 50 µm (b), 25 µm (e)

Myofibers are broadly divided into slow‐twitch (type I) and fast‐twitch (type II) fibers. The size of slow‐twitch fibers is less affected by aging compared to fast‐twitch fibers (Lexell, 1995; Nilwik et al., 2013). Therefore, soleus muscle, which is predominantly composed of slow‐twitch fibers, is more resistant to age‐related atrophy, while muscles with fast‐twitch fiber predominance are more prone to atrophy (Figure 4a) (Deschenes et al., 2013). Interestingly, slow‐twitch soleus muscle is also more resistant to age‐related NMJ denervation than fast‐twitch EDL muscle (Chai et al., 2011). Because TA muscle is also predominantly composed of fast‐twitch fibers with very few slow‐twitch fibers (Ham et al., 2020; Motohashi et al., 2019), TA muscle was used as fast‐twitch muscle in addition to EDL muscle in this study. Similarly to the previous study (Chai et al., 2011), slow‐twitch soleus muscle exhibited resistance to age‐related NMJ denervation, which is prominent in fast‐twitch TA muscle (Figure 5a). We next compared MFG‐E8 protein levels at NMJs between slow‐twitch soleus muscle and fast‐twitch TA muscle in aged mice. Intriguingly, aged soleus muscle showed significantly lower levels of MFG‐E8 at NMJs (Figure 5b). The signal intensity of MFG‐E8 in NMJs of aged soleus muscle was significantly lower than that of aged TA muscle (Figure 5c,d). Association between low levels of MFG‐E8 at NMJs and resistance to denervation in soleus muscle conversely suggests a functional involvement of increased MFG‐E8 in age‐related denervation. To determine the association between arterial MFG‐E8 and sarcopenic phenotype, we examined age‐related changes of arterial MFG‐E8 in sarcopenia‐resistant soleus muscle. A significant increase in MFG‐E8 protein was observed in the arterial inner wall of aged soleus muscle (Figure 5e), indicating that the increase in MFG‐E8 at arteries is not related to sarcopenic phenotype.

### 2.6 Absence of MFG‐E8 attenuates age‐related denervation of NMJs and muscle weakness

**FIGURE 6:**
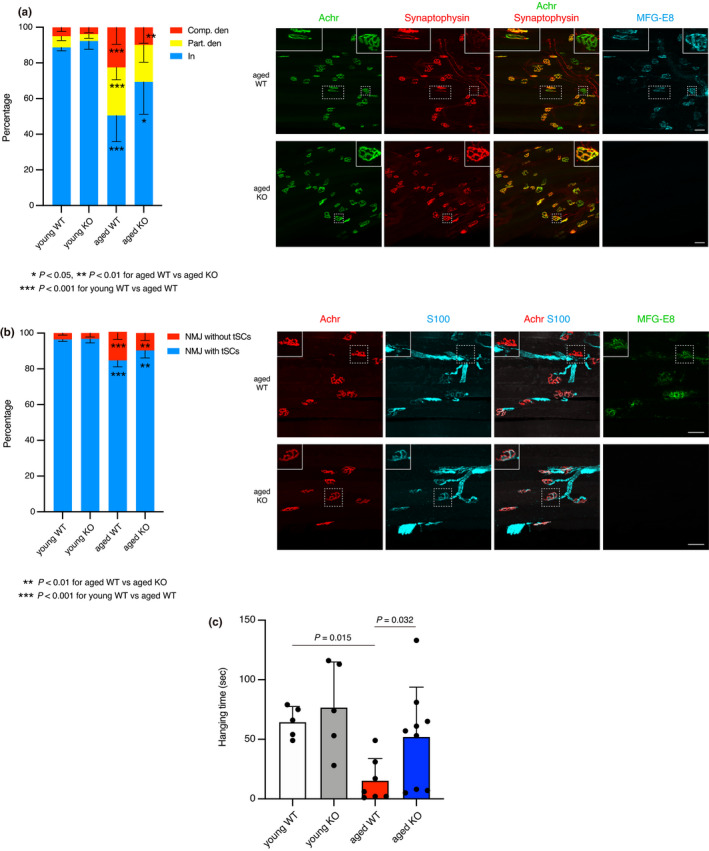
Absence of MFG‐E8 attenuates age‐related denervation of NMJs and loss of muscle function. (a) Ratios of completely (Comp. den) and partially (Part. den) denervated, and innervated (In) NMJs in EDL muscle. Data represent the means ± *SD*; ANOVA followed by Fisher's LSD tests; *p < 0.05, **p < 0.01, for aged WT vs. aged KO. ***p < 0.001, for young WT vs. aged WT. Whole‐mount immunostaining of EDL muscles of aged WT and MFG‐E8 KO mice (aged 21 months) for Achr, synaptophysin, and MFG‐E8. Insets show magnified images of boxed regions. Left and right insets in upper panels show partially (Part. den) and completely (Comp. den) denervated NMJ, respectively. Insets in lower panels show innervated (In) NMJ. (b) Ratios of NMJs without or with terminal Schwann cells (tSCs) in TA muscle. Data represent the means ± *SD*; ANOVA followed by Fisher's LSD tests; **p < 0.01, for aged WT vs. aged KO. ***p < 0.001, for young WT vs. aged WT. Immunostaining of TA muscles of aged WT and MFG‐E8 KO mice (aged 21 months) for Achr, S100, and MFG‐E8. Insets show magnified images of boxed regions. Insets in upper panels show NMJ without tSCs. Insets in lower panels show NMJ with tSCs. (c) Length of time spent hanging in Wire Hanging Test Chamber. Data represent the means ± *SD*; ANOVA followed by Fisher's LSD tests. In both genotypes, mice aged 8–11 months and aged 20–22 months were used as young and aged mice, respectively. *n* = 5 for young WT and KO, *n* = 7 for aged WT, *n* = 9 for aged KO. Scale bars: 50 µm (a, b)

We directly investigated the effects of increased levels of MFG‐E8 on age‐related denervation using MFG‐E8 knockout (KO) mice. Deficiency of MFG‐E8 was confirmed in both mRNA and serum MFG‐E8 levels (Figure S1A,B). Fast‐twitch EDL or TA muscles from KO mice and wild‐type (WT) littermates were subjected to the analyses for NMJ status. Young KO and WT mice showed comparable NMJ status with most NMJs being normally innervated (Figure 6a). While WT mice showed age‐related denervation as observed in the time course study (Figure 4), aged KO mice exhibited well preserved NMJs (Figure 6a). When compared to aged WT mice, aged KO mice completely lacked MFG‐E8 at NMJs and had more innervated and less denervated NMJs (Figure 6a). We next examined the effect of MFG‐E8 deficiency on age‐related changes of tSCs. Consistent with previous study (Snyder‐Warwick et al., 2018), the ratio of NMJs covered by tSCs decreased with age (Figure 6b). Interestingly, the ratio of NMJs with tSCs in aged KO mice was significantly higher than that of aged WT mice (Figure 6b), indicating the adverse effect of increased MFG‐E8 on tSCs. Reflecting the healthier NMJ status of aged KO mice, wire hanging tests revealed that hanging time of aged KO mice was significantly longer than those of aged WT mice even though body weights did not differ between genotypes (Figure 6c and Figure S2), indicating the preserved muscle function in aged KO mice. On the other hand, the loss of muscle weight occurred in muscles of KO mice at a similar level to WT mice except for sarcopenia‐resistant soleus muscle (Figure S2). Collectively, these results indicate that the increase of MFG‐E8 protein at NMJs is an exacerbating factor for age‐related NMJ degeneration and muscle weakness although it is not associated with loss of muscle mass with age.

## 3 DISCUSSION

The amount of MFG‐E8 significantly increased in the arteries and NMJs of skeletal muscles with aging. Using MFG‐E8 KO mice, we showed that MFG‐E8 deficiency alleviates age‐related NMJ denervation and loss of muscle strength. Therefore, MFG‐E8 is likely to be functionally involved in skeletal muscle aging and could be a promising therapeutic molecular target for the prevention of sarcopenia.

The mechanisms leading to age‐related denervation of NMJs are not completely understood. Unlike myelinating SCs that surround the axons of motor nerves, tSCs are non‐myelinating glial cells that cover NMJs. Aging leads to the degeneration of tSCs (Ludatscher et al., 1985) and a decrease in both the number of tSCs and the ratio (%) of NMJs that they cover (Snyder‐Warwick et al., 2018). Because tSCs are necessary for the formation, maintenance, and regeneration of NMJs (Barik et al., 2016; Li et al., 2018), age‐related tSC deterioration might contribute to NMJ denervation. We revealed that MFG‐E8 protein is conspicuously abundant on the surface of tSCs in aged NMJs, and that a genetic deficiency of MFG‐E8 attenuated NMJ denervation during aging. Although we examined apoptosis of tSCs, we could not see any signals for activated caspase 3 around aged NMJs (Figure S3). These results suggested that increased levels of MFG‐E8 lead to NMJ denervation through the dysregulation of tSCs not through death of tSCs. Although the lack of specific markers of tSCs interferes with the ability to study this cell type, analyzing the effect of increased MFG‐E8 on tSCs might deepen our understanding of the mechanisms underlying age‐related NMJ degeneration.

The role of MFG‐E8 appears to be context‐dependent. During myocardial infarction, MFG‐E8 secreted from myofibroblasts facilitates recovery by promoting apoptotic engulfment (Nakaya et al., 2017). In contrast, increased MFG‐E8 is closely associated with neuromuscular diseases. Patients with amyotrophic lateral sclerosis (ALS) have higher levels of MFG‐E8 in cerebrospinal fluid, and these correlate with ALS severity (Yang et al., 2020). Aggregates of MFG‐E8/medin in the aorta and brain vasculature cause cerebrovascular dysfunction in aged mice (Degenhardt et al., 2020). Cerebrovascular MFG‐E8/medin is also associated with Alzheimer's disease (AD) and vascular dementia, indicating that MFG‐E8/medin could be a novel risk factor or biomarker for AD and vascular dementia (Migrino et al., 2020). Here, we revealed age‐related increase in MFG‐E8 levels at NMJs, which eventually led to NMJ denervation and muscle weakness. More MFG‐E8 notably accumulated at NMJs in fast‐twitch, than slow‐twitch muscles. The effects of aging are more severe in fast‐twitch than in slow‐twitch muscle (Deschenes et al., 2013; Nilwik et al., 2013), and this is also true in terms of the age‐related degeneration of NMJs. In fact, aging NMJs become severely denervated in fast‐twitch muscles, whereas NMJs remain innervated in slow‐twitch muscles (Chai et al., 2011; Deschenes et al., 2010). The correlation between MFG‐E8 levels and muscle type‐dependent susceptibility with sarcopenia further supports the notion that increased MFG‐E8 is an exacerbating factor of age‐related NMJ degeneration and muscle weakness.

The content of MFG‐E8 mRNA and protein increases in the arterial walls of aged, atherosclerotic, hypertensive, and diabetic mammals, including humans (Fu et al., 2009; Wang et al., 2013). Overexpressed MFG‐E8 in vitro induces endothelial cell apoptosis (Li et al., 2011; Li et al., 2011). Furthermore, incubating vascular smooth muscle cells with MFG‐E8 in vitro increases invasion capacity, proliferation, and expression of proinflammatory genes, such as NF‐κB, indicating arterial inflammation (Chiang et al., 2019; Fu et al., 2009; Wang et al., 2012). Hence, MFG‐E8 is considered as a key molecule in the pathogenesis of chronic arterial inflammation (Wang et al., 2013, 2014). Arterial amyloidosis occurs mainly in the upper body of virtually all individuals aged >50 years (Mucchiano et al., 1992; Peng et al., 2005), and medin forms fibrils of arterial amyloid (Häggqvist et al., 1999; Peng et al., 2005). Degenhardt et al. recently revealed that a genetic deficiency of MFG‐E8 eliminates vascular aggregates and prevents the age‐related decline of cerebrovascular function in mice, suggesting that age‐related MFG‐E8/medin aggregation causes cerebrovascular dysfunction (Degenhardt et al., 2020). Here, we found increased levels of MFG‐E8 protein at NMJs in skeletal muscle as well in the vasculature. A genetic deficiency of MFG‐E8 attenuated NMJ denervation and the decline of muscle strength in aged mice, providing direct evidence of a pathogenic role of MFG‐E8 in age‐related sarcopenia. To distinguish medin from MFG‐E8 using antibodies is difficult because medin is an internal fragment of MFG‐E8. Analysis of proteins extracted from amyloid‐rich thoracic aortic media in humans revealed the presence of both MFG‐E8 and medin (Peng et al., 2005). Furthermore, although Western blots of aged mouse aortic samples revealed medin (~5 kDa) and fragments containing medin (~17 kDa), bands of full‐length MFG‐E8 were more intense than those of medin (Degenhardt et al., 2020). The present study did not find bands corresponding to medin in Western blots (Figure 1b). Therefore, further studies are required to clarify which form of MFG‐E8 exerts more adverse effects.

Using MFG‐E8 KO mice, we revealed that increased MFG‐E8 protein is not involved in age‐related loss of muscle mass. In the aging process, the loss of muscle strength occurs more rapidly than the loss of muscle mass (Delmonico et al., 2009). Epidemiological data indicate that low muscle strength is strongly associated with adverse outcomes, such as long hospital stays, increased functional limitations, poor health‐related quality of life, and death (Cruz‐Jentoft et al., 2019). Therefore, muscle strength is a more important indicator than muscle mass. Although increased MFG‐E8 protein was not related to age‐related loss of muscle mass, aged MFG‐E8 KO mice exhibited alleviated NMJ denervation and muscle weakness. Therefore, this study provides a rationale for the therapeutic concept that lowering MFG‐E8 levels is beneficial for maintaining muscle health.

In conclusion, we found an age‐related increase in MFG‐E8 levels at NMJs and revealed its pathogenic role in skeletal muscle deterioration by aging. Our study suggests that targeting MFG‐E8 may be a novel therapeutic approach to prevent sarcopenia and sustain healthy aging.

## 4 EXPERIMENTAL PROCEDURES

### 4.1 Mice

From 2‐ to 3‐month‐old (young) C57BL/6 mice were purchased from Japan SLC. C57BL/6 mice aged >6months were originally obtained from Japan SLC and maintained in the laboratory animal facility of Tokyo Metropolitan Institute of Gerontology (TMIG). MFG‐E8 KO mice (Hanayama et al., 2004) (RBRC01726) were originally obtained from RIKEN BRC. Background strain of MFG‐E8 KO mice is C57BL/6. Heterozygous mice were mated to generate KO and WT littermates and maintained in the laboratory animal facility of TMIG until they were subjected to analyses.

### 4.2 Cytokine antibody array

Total protein was extracted from the TA muscles of two young and two aged mice. TA muscles were lysed with 10 mM Tris‐HCl (pH 7.5) containing 150 mM NaCl, 1% sodium deoxycholate, 1% TritonX‐100, and protease inhibitor cocktail (Roche). The lysates were sonicated for 5 min, centrifuged at 18,000× g for 15 min at 4°C, then protein concentrations were determined in supernatants using BCA assays (Thermo Fisher Scientific). Lysates (100 µL) containing 150 µg of protein were incubated at 4°C overnight with a RayBio Mouse Cytokine Antibody Array G Series 2000 (Ray Biotech) as described by the manufacturer. Images were acquired using a GenePix 4000B Scanner (Molecular Devices), and signal intensity was imported into Array‐Pro Analyzer Ver.4.5 (Media Cybernetics) and analyzed at Filgen.

### 4.3 ELISA

Total protein was extracted from the TA muscles of four young and four aged mice, as described above. Levels of MFG‐E8 in lysates (50 µl) containing 30 µg of protein were determined using Mouse MFG‐E8 Quantikine ELISA Kit (R&D Systems) as described by the manufacturer.

### 4.4 Immunoblotting

Total protein was extracted from the TA muscles of four young and four aged mice, as described above. Portions of lysates containing 30 µg of protein or mouse recombinant MFG‐E8 (R&D Systems) were separated by 10−20% SDS‐polyacrylamide gel (Wako) electrophoresis and transferred onto PVDF membranes (Pall Corporation). After blocking‐nonspecific binding with 1% skim milk, the membranes were probed with 1:1,000‐diluted anti‐MFG‐E8 (R&D Systems) or 1:1000‐diluted anti‐GAPDH (clone 14C10; Cell Signaling Technology) antibodies. The membranes were washed with Tris‐buffered saline, 0.1% Tween‐20 (TBST), then incubated with 1:5,000‐diluted alkaline phosphatase‐conjugated anti‐goat IgG (Jackson ImmunoResearch) for MFG‐E8 or 1:1000‐diluted anti‐rabbit IgG (Dako) for GAPDH. Signals were detected using 5‐bromo‐4‐chloro‐3‐indolyl phosphate/nitro blue tetrazolium (Sigma‐Aldrich).

### 4.5 RNA extraction and quantitative PCR

Total RNA was extracted from the TA muscles of nine young and nine aged mice using miRNeasy Mini Kit (Qiagen), and equal amounts of RNA were reverse transcribed into cDNA using QuantiTect Reverse Transcription Kit (Qiagen). Real‐time quantitative PCR proceeded on a Thermal Cycler Dice Real Time System (Takara Bio) with SYBR Premix Ex Taq II (Takara Bio) at 94°C for 30 s, followed by 40 cycles of 94°C for 5 s, 60°C for 20 s, 72°C for 10 s, and dissociation curve analysis. Cytidine monophospho‐N‐acetylneuraminic acid synthetase (*Cmas*) was the internal control gene for quantifying gene expression in mouse tissues (Yoshimoto et al., 2020; Zhao & Hoffman, 2004).

The specific forward and reverse (5′→3′) PCR primer sequences were as follows: *Mfge8*: AAGGAATGGCTGCAGGTTGAC and ACTGCACACCATCATCACTGTG; *Cmas*: CAAAGGCATCCCACTGAAGA and CCCACACACTCTGGAAGACC.

### 4.6 Immunohistochemistry and microscopy

Fresh muscle samples of mice were rapidly frozen in isopentane cooled with liquid nitrogen. For the observation of NMJ, muscles were fixed in 2% PFA for 30 min, immersed in 20% sucrose, and frozen. Thereafter, 40‐µm‐thick longitudinal sections were cut using a cryostat and incubated with 1 µg/ml of α‐bungarotoxin (Invitrogen) for 1 h. The sections were washed with PBS and immersed in methanol for 5 min at −20°C. For the observation of human MFG‐E8 in NMJ, 20‐µm‐thick longitudinal sections were heated with antigen retrieval reagent (Basic, R&D Systems) for 5 min at 95°C. For the observation of arteries, 7‐µm‐thick transverse sections of fresh frozen muscles were made. Fresh frozen sections were fixed with 4% PFA for 5 min and washed with PBS. For the observation of human MFG‐E8, the sections were heated with antigen retrieval reagent for 5 min at 95°C. Non‐specific protein binding was blocked by incubating specimens with Protein Block Serum‐Free reagent (Dako) for 10 min, then the specimens were incubated overnight at 4°C with the following primary antibodies: 1:1000‐diluted Armenian hamster anti‐mouse MFG‐E8 (clone 18A2‐G10; MBL), 1:500‐diluted rabbit anti‐human MFG‐E8 (Sigma‐Aldrich), and 1:400‐diluted Cy3‐conjugated α‐smooth muscle actin (clone 1A4; Sigma‐Aldrich); 1:200‐diluted rat anti‐mouse CD31 (clone 390; BioLegend); 1:100‐diluted mouse anti‐human CD31 (clone WM59; BioLegend); 1:200‐diluted rabbit anti‐synaptophysin (Abcam), 1:2‐diluted rabbit anti‐S100 (Dako), and 1:200‐diluted mouse anti‐SV2 (DSHB). The specimens were washed and stained for 1 h with the following secondary antibodies diluted 1:1000: Alexa Fluor 488‐, Cy3‐, or Alexa Fluor 647‐conjugated goat anti‐Armenian hamster IgG, Alexa Fluor 488‐, Cy3‐, or Alexa Fluor 647‐conjugated donkey anti‐rabbit IgG, Cy3‐conjugated donkey anti‐rat IgG, and Cy3‐conjugated donkey anti‐mouse IgG (all from Jackson ImmunoResearch). Specimens were counterstained with DAPI (Dojindo) and mounted with SlowFade Diamond antifade reagent (Invitrogen). In indicated experiments, muscle sections were processed for immunofluorescence analysis and then stained with hematoxylin and eosin (HE). Images were acquired using a BX50 fluorescence microscope equipped with a DP70 CCD camera (both from Olympus), confocal laser scanning microscope system LSM700 (Carl Zeiss), or TCS SP8 (Leica).

### 4.7 Whole‐mount immunofluorescence staining

The EDL muscle was split into four pieces, fixed in 2% PFA for 30 min, and then washed with PBS. Non‐specific binding was blocked with 1% Triton X‐100 containing 4% BSA in PBS (blocking solution) at 4°C overnight, then the muscle specimens were incubated at 4°C for 1 d with the antibodies diluted in blocking solution. Thereafter, the muscles were incubated with secondary reagents diluted in blocking solution at 4°C for 1 day. Stained muscles were mounted with SlowFade Diamond Antifade Reagent (Invitrogen). Z‐stack images were acquired using the LSM 700 confocal laser scanning microscope system, and reconstructed images were visualized by shadow projection using ZEN software (Carl Zeiss).

### 4.8 Assessment of innervation at NMJs and quantification of MFG‐E8 signal intensity

Z‐stack images of whole‐mount immunofluorescence stained EDL, 40‐µm‐thick longitudinal TA, and soleus sections, and cross‐sections of human gluteus medius were acquired using the TCS SP8 confocal laser scanning microscope system (Leica), and reconstructed images were visualized by maximum intensity projection using LAS X software (Leica). For denervation assessment, we analyzed in 50–100 NMJs of EDL muscle per mouse, 20–60 NMJs of TA muscle per mouse, 10–50 NMJs of soleus muscle per mouse. Numbers of completely or partially denervated, and innervated NMJs were counted. For quantification of MFG‐E8 signal intensity, we analyzed in 20–80 NMJs of TA muscle per mouse, 10–50 NMJs of soleus muscle per mouse, 6–17 arteries of gluteus medius per human. The signal intensity of MFG‐E8 was quantified using ImageJ software (NIH). The region of interest (ROI) was manually set to NMJs and arteries, and then, the intensity of the fluorescence signal of MFG‐E8 was measured.

### 4.9 Human muscle samples

#### 4.9.1 NMJ analysis

We obtained muscle samples for NMJ analysis from amputated lower limb of three patients (age 64: male, age 90: female, and age 78: male) at the TMIG. The direct cause of lower limb amputation is necrosis associated with lower extremity artery disease caused by diabetes or ulcers. TA or GC muscles (5–10 cm long) were obtained from surgical specimens immediately after lower limb amputation. Samples were harvested from the proximal end near the surgical incision line in areas showing good bleeding and muscle fasciculation away from areas of necrosis. Muscle blocks cut to a length of ~1 cm were fixed in 2% paraformaldehyde (PFA) for 1 h, immersed in 20% sucrose, and frozen.

#### 4.9.2 Artery analysis

For artery analysis, small muscle fragments of the gluteus medius were obtained during total hip arthroplasty from five young‐middle (age 30: male, age 46: male, age 47: male, age 38: female, age 48: female) and five aged (age 73: female, age 75: female, age 76: female, age 79: female, age 85: female) patients at Fujita Health University. Muscle samples were immediately frozen in isopentane cooled with liquid nitrogen.

### 4.10 Wire hanging test

The amount of time that mice gripped a wire mesh before they let go was measured using a wire hanging test chamber (O’Hara & Co., Ltd.).

### 4.11 Statistics

Data were statistically analyzed using GraphPad Prism 8 (GraphPad Software). No statistical methods were used to predetermine the sample size. Body weight, muscle weight, and wire hanging time measurements were measured in a blinded manner; other experiments were not blinded. Two‐tailed unpaired Student's *t* tests were used for comparison between two groups. For comparisons of more than two groups, one‐way analysis of variance (ANOVA) followed by Dunnett's tests or Fisher's LSD tests was used.

### 4.12 Study approval

Experiments using mice were approved by the Experimental Animal Care and Use Committee of Tokyo Metropolitan Geriatric Hospital and Institute of Gerontology. Experiments using human samples were approved by the Ethical Committee at Tokyo Metropolitan Geriatric Hospital and Institute of Gerontology, and Fujita Health University. All subjects provided written informed consent.

## CONFLICT OF INTEREST

The authors declare that they have no conflict of interest.

## AUTHOR CONTRIBUTION

M.I‐U. conceived the study, designed and conducted experiments, analyzed data, and wrote the manuscript. A.U. assisted with immunohistochemical experiments and manuscript writing. H.Z. and Y.Y. maintained the mouse models. H.Z. assisted with ELISA experiments. T.K. and A.U. assisted with the RNA experiments. M.T., N.K., T.N., M.M., and K.T. provided human muscle tissues. M.I‐U. and A.U. interpreted the results and coordinated the project.

## Supporting information

Figure S1‐S3

Table S1

## Data Availability

The data that support the findings of this study are available from the corresponding author upon reasonable request.
