# Supplementary material for: Increased MFG‐E8 at neuromuscular junctions is an exacerbating factor for sarcopenia‐associated denervation
Source: Aging Cell. 2021 Dec 24;21(1):e13536. doi: 10.1111/acel.13536 (PMC8761010; doi:10.1111/acel.13536)
Supplement: Supplementary file 1 — Figure S1‐S3 [file ACEL-21-e13536-s002.pdf]

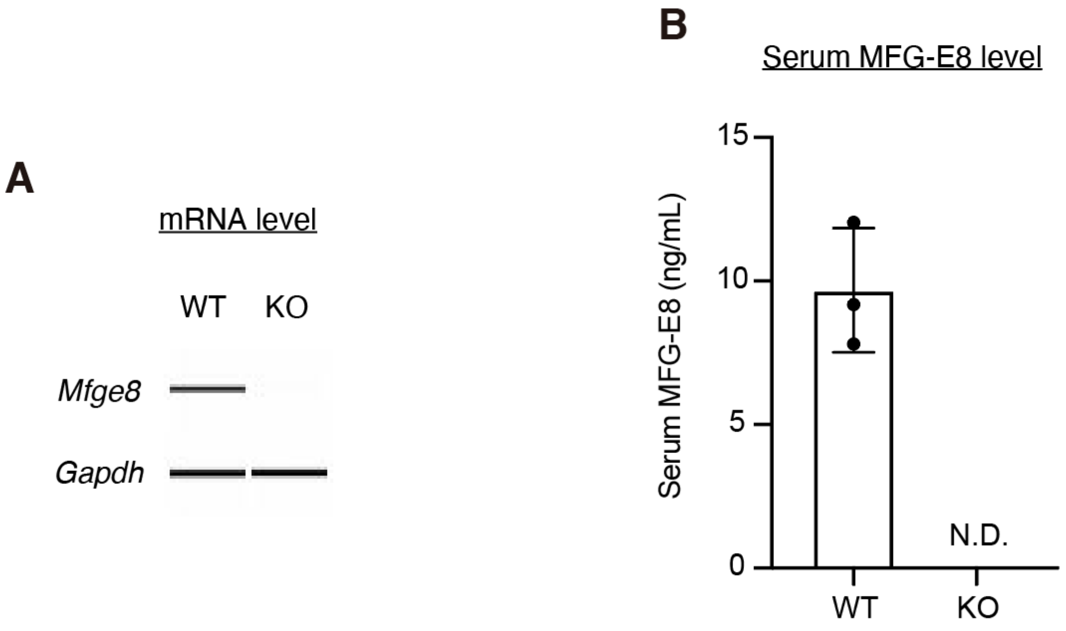

Supplemental Figure 1. Deficiency of *Mfge8* mRNA and serum MFG-E8 protein in MFG-E8 KO mice

(A) Deficiency of *Mfge8* mRNA in TA muscle of MFG-E8 KO mouse. PCR proceeded on a Thermal Cycler (Eppendorf) with Ex Taq (Takara Bio) at 94°C for 2 m, followed by 24 cycles of 94°C for 30 s, 60°C for 30 s, 72°C for 1 m, and finally at 72°C for 2 m. PCR products were analysed by Microchip Electrophoresis System (MultiNA, Shimadzu). Internal control is *Gapdh*. The specific forward and reverse (5′→3′) PCR primer sequences are:

Mfge8: AAGGAATGGCTGCAGGTTGAC and ACTGCACACCATCATCACTGTG; *Gapdh*: CCTGGAGAAACCTGCCAAGTATG and AGAGTGGGAGTTGCTGTTGAAGTC. Female MFG-E8 WT and KO mice (aged 22 months). (B) Deficiency of MFG-E8 protein in serum of MFG-E8 KO mice. MFG-E8 serum levels were analysed by ELISA. Female MFG-E8 WT and KO mice (aged 18-19

months). n = 3 per group. Data represent the mean ± SD. N.D.; not detectable.

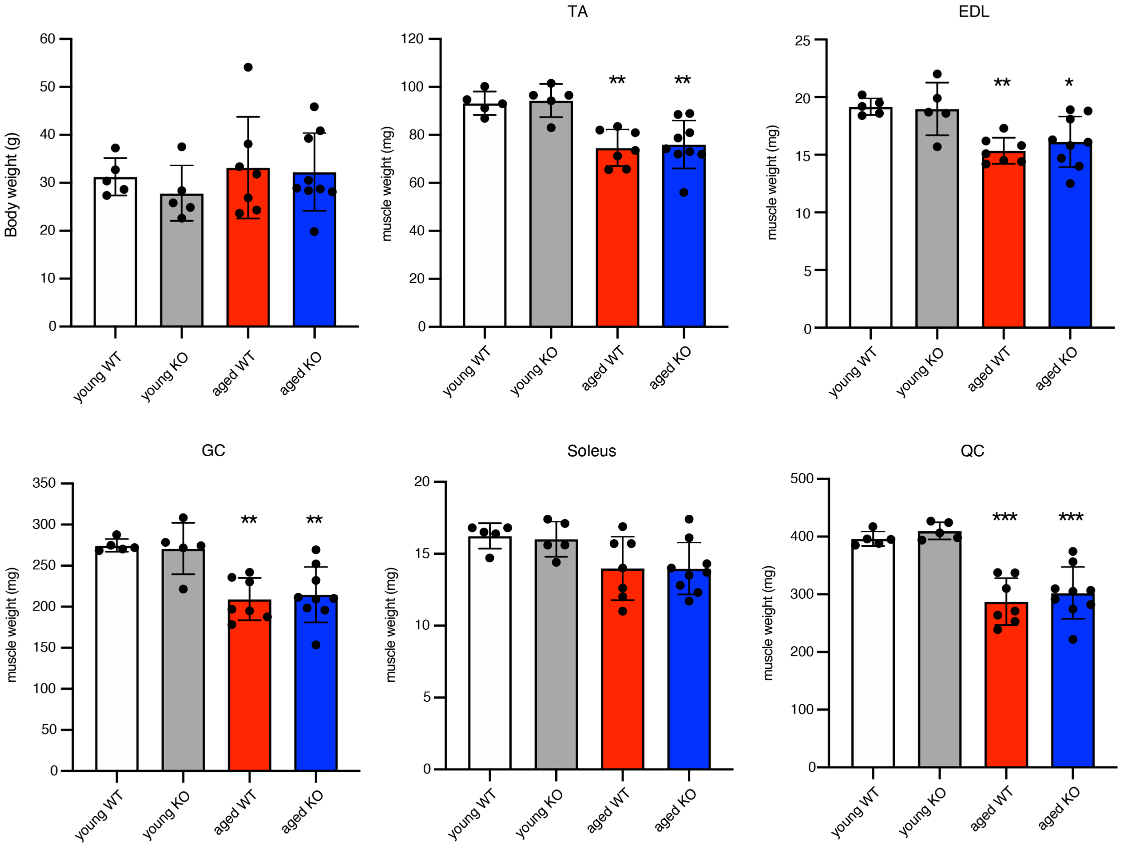

Supplemental Figure 2. Body weight and muscle weight of young and aged WT and MFG-E8 KO mice

Female young MFG-E8 WT and KO mice (aged 8-11 months). Female aged MFG-E8 WT and KO mice (aged 20–22 months). n = 5 for young WT and KO. n = 7 for aged WT. n = 9 for aged KO. Data represent the mean ± SD; ANOVA followed by Dunnett's test; **P* < 0.05, ***P* < 0.01, *** *P* < 0.001 vs young WT. TA, tibialis anterior; EDL,

extensor digitorum longus; GC, gastrocnemius; QC, quadriceps.

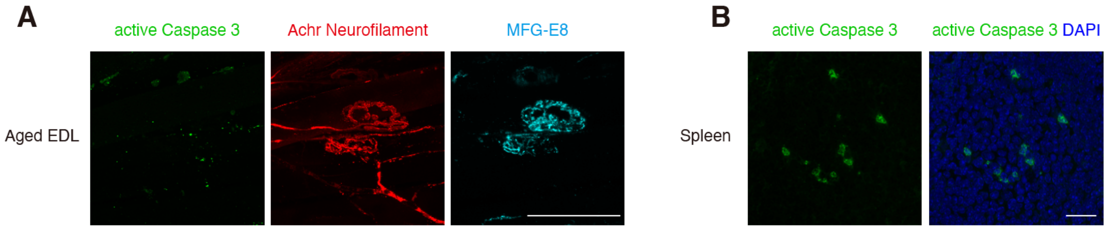

Supplemental Figure 3. Apoptosis is not detected in NMJ of aged mice.

(A) Whole-mount immunostaining of EDL muscle of female aged mouse (aged 20 months) for active caspase 3, Achr, Neurofilament, and MFG-E8. Rabbit anti-Caspase 3 antibody (1:50; Sigma-Aldrich), mouse anti-neurofilament antibody (1:1,000; clone SMI312; BioLegend). (B) Positive control staining of active caspase 3 in spleen of male mouse (aged 9 months). Scale bars: 75 µm (A), 25 µm (B).
