# Supplementary material for: Increased MFG‐E8 at neuromuscular junctions is an exacerbating factor for sarcopenia‐associated denervation
Source: Aging Cell. 2021 Dec 24;21(1):e13536. doi: 10.1111/acel.13536 (PMC8761010; doi:10.1111/acel.13536)
Supplement: Supplementary file 2 — Table S1 [file ACEL-21-e13536-s001.pdf]

Supplemental Table 1. Muscles used and sex in each experiment

Figure or Table number	Experiment	Muscle used	sex
Table 1	cytokine antibody array	TA	male
Figure 1A	ELISA	TA	male
Figure 1B	immunoblot	TA	male
Figure 1C	real-time PCR	TA	male, n = 4; female, n = 5 (both young and aged)
Figure 2A, B	immunohistochemistry	TA	male
Figure 2C	whole-mount immunostaining	EDL	male
Figure 2D, E	Quantification of MFG-E8 signal intensity in immunohistochemistry images	TA	male, n = 3; female, n = 2 (both young and aged)
Figure 2F	immunohistochemistry	TA	male
Figure 3A, B	immunohistochemistry	gluteus medius (human)	female
Figure 3C	Quantification of MFG-E8 signal intensity in immunohistochemistry images	gluteus medius (human)	male, n = 3; female, n = 2 (young)<br>female, n = 5 (aged)
Figure 3D	immunohistochemistry	TA (human)	female
Figure 4A	Measurement of muscle weight	TA, EDL, Soleus, GC, QC	female
Figure 4B	Quantification of denervation rate in immunohistochemistry images	TA	female
Figure 4C	Quantification of MFG-	TA	female

	E8 signal intensity in immunohistochemistry images		
Figure 5A	Quantification of denervation rate in immunohistochemistry images	Soleus, TA	male, n = 3; female, n = 3 (both soleus and TA)
Figure 5B	immunohistochemistry	Soleus, TA	female
Figure 5C, D	Quantification of MFG-E8 signal intensity in immunohistochemistry images	Soleus, TA	male, n = 3; female, n = 3 (both soleus and TA)
Figure 5E	immunohistochemistry	Soleus	male
Figure 6A	Immunohistochemistry, Quantification of denervation rate in whole-mount immunostaining images	EDL	female
Figure 6B	immunohistochemistry, Quantification of rate of NMJ without tSCs in immunohistochemistry images	TA	female

TA; tibialis anterior

EDL; extensor digitorum longus

GC; gastrocnemius

QC; quadriceps

tSCs; terminal Schwann cells
